# MRI-based cell tracking of OATP-expressing cell transplants by pre-labeling with Gd-EOB-DTPA

**DOI:** 10.21203/rs.3.rs-3698429/v1

**Published:** 2023-12-12

**Authors:** Tapas Bhattacharyya, Christiane Mallett, Erik M Shapiro

**Affiliations:** Michigan State University; Michigan State University; Michigan State University

**Keywords:** MRI, cell transplant, gadolinium, OATP1B3, cardiac

## Abstract

**Purpose:**

A critical step in cell-based therapies is determining the exact position of transplanted cells immediately post-transplant. Here, we devised a method to detect cell transplants immediately post-transplant, using a clinical gadolinium-based contrast agent. These cells were detected as hyperintense signals using a clinically familiar T1-weighted MRI protocol.

**Procedures:**

HEK293 cells were stably transduced to express human OATP1B3, a hepatic organic anion transporting polypeptide that transports Gd-EOB-DTPA into cells that express the transporters, the intracellular accumulation of which cells causes signal enhancement on T1-weighted MRI. Cells were pre-labeled prior to injection in media containing Gd-EOB-DTPA for MRI evaluation and indocyanine green for cryofluorescence tomography validation. Labeled cells were injected into chicken hearts, in vitro, after which MRI and cryofluorescence tomography were performed in sequence.

**Results:**

OATP1B3-expressing cells had substantially reduced T1 following labeling with Gd-EOB-DTPA in culture. Following their implantation into chicken heart, these cells were robustly identified in T1-weighted MRI, with image-derived injection volumes of cells commensurate with intended injection volumes. Cryofluorescence tomography showed that the areas of signal enhancement in MRI overlapped with areas of indocyanine green signal, indicating that MRI signal enhancement was due to the transplanted cells.

**Conclusions:**

OATP1B3-expressing cells can be pre-labeled with Gd-EOB-DTPA prior to injection into tissue, affording the use of clinically familiar T1-weighted MRI to robustly detect cell transplants immediately after transplant. This procedure is easily generalizable and has potential advantages over the use of iron oxide based cell labeling agents and imaging procedures.

## Introduction

Non-invasive imaging can play a key role in clinical cell therapies by determining the precise locations of transplanted cells immediately post-cell transplant. MRI-based cell tracking, as opposed to nuclear imaging methods, is most useful when the high-resolution and spatial discrimination capabilities of MRI are used for localization of transplanted cells and providing soft tissue anatomical context. Most commonly, iron oxide nanoparticles have been used for magnetic cell labeling with detection of labeled cells via T2/T2*-weighted MRI^1^, with sensitivities as low as single cells^2^ and even single particles^3^. However, two problems have plagued iron oxide-based MRI-based cell tracking since its inception: 1) the T2/T2*-weighted dark contrast obscures the underlying anatomy, making quantification of cell number difficult, and 2) the iron oxide nanoparticles can still create MRI contrast even if the original labeled cell is long dead. Bright contrast methods have been used occasionally for MRI-based cell tracking (excellent review in ^4^) employing Gd-chelates and Mn^2+^, accumulated intracellularly by disruptive (photoporation, transfection, etc) methods or by simple incubation, respectively. Further, ^19^F agents have been used for MRI-based cell tracking^5^. Yet, MRI-based cell tracking has not made significant clinical impact, partly due to the complications of cell labeling and detection.

Hepatic organic anion transporting polypeptides (**OATPs**) are a new category of MRI reporter proteins^6–12^. OATPs are ~ 700 amino acid membrane proteins with 12 membrane-spanning helices, whose expression is conserved among vertebrates ^13^. Hepatic OATPs transport off-the-shelf, FDA-approved, clinically used, MRI contrast agents into cells ^14–15^. In clinical scenarios, following IV injection of Gd-EOB-DTPA or Gd-BOPTA, two FDA-approved hepatospecific MRI contrast agents, hepatocytes become hyperintense on T1-weighted MRI due to the intracellular accumulation of the Gd-based contrast agent. This is used clinically to detect tumors in the liver as tumors (generally) do not express OATPs and remain hypointense in relation to the bright liver ^16^. As it relates to the use of OATPs as MRI reporter proteins, OATPs with reported efficient transport of Gd-EOB-DTPA include human/primate OATP1B1 and OATP1B3 ^14^, rat OATP1A1 ^8, 11^, and rat OATP1B2 ^17^. Many other species, including mouse ^18–20^, rabbit ^21^, dog ^22–24^ and pig ^25^, exhibit hepatic accumulation of Gd-EOB-DTPA and Gd-BOPTA, as evidenced by liver MRI, so it is likely that there are other members of the hepatic OATP1B family (dog OATP1B4, e.g.) that transport these agents as well.

To date, the use of hepatic OATPs as reporter proteins has relied on the intravenous injection of Gd-EOB-DTPA or Gd-BOPTA to accumulate in cells post-transplant ^6–12^. We hypothesized that OATP1B3-expressing cells can be pre-labeled with Gd-EOB-DTPA prior to injection affording the use of clinically familiar T1-weighted MRI to robustly detect cells immediately post-transplantation, similarly to other cell labeling paradigms. This straightforward approach to labeling and MRI detection may facilitate the incorporation of MRI-based cell tracking in clinical trials and cell therapies.

## Materials and methods

Lentivirus encoding human OATP1B3 (custom designed and ordered from Vectorbuilder) was used for stable transduction in mammalian cells. HEK293 cells (ATCC) were infected at MOI 10:1 and underwent 3-week antibiotic selection to create stably expressing cells. Stable transgene expression was verified by RT-qPCR. Total RNA was isolated from both HEK293 (wild type/untransduced cells) and HEK-OATP1B3 (transduced /OATP1B3 stable) cell line by PureLink RNA Mini Kit (Invitrogen). cDNA was synthesized by using RevertAid First Strand cDNA Synthesis Kit (Thermo Fisher Scientific) TaqMan probe-based primer for Human OATP1B3 (Hs00251986_m1) and human GAPDH (hs02786624), reference gene were purchased from Thermo Fisher Scientific. PCR was carried out in CFX96 (Biorad) real time thermal cycler. cDNA from wild type HEK293 and transduced HEK-OATP1B3 were used as a template for the quantitative PCR for OATP1B3 and GAPDH (reference gene). OATP1B3 expression was normalized by GAPDH expression in both the cell line. Total increase in OATP1B3 expression in transduced cell line in comparison to the wild type cells was measured by DDCq method. The average ΔCq for the untransduced HEK293 cells was subtracted from all the ΔCq values to determine ΔΔCq. Relative fold of change in the OATP1B3 gene expression was calculated by 2^-(ΔΔCq).

Expression of OATP1B3 in the transduced cells were also verified by standard western blot protocol. RIPA buffer (Thermo scientific) with 1X-Halt Protease inhibitor (Thermo Scientific) was used as a lysis buffer. Cells were lysed by repeated cycles (10s) of sonication followed by keeping on ice for 50 seconds. Total protein concentration was measured by using BCA protein estimation kit (Themo Scientific). Proteins were resolved by SDS PAGE in precast Mini-PROTEIN TGX (4–20%) GEL (Biorad). Resolved proteins from the gel was transferred to a nitrocellulose membrane by using iBlot2 (Invitrogen). After the transfer, membrane was allowed to dry completely before blocking step. The membrane was blocked by incubating in a shaking platform for 1h at room temperature with 4% nonfat powdered milk in 1X Tris Buffered Saline (TBS, Fisher Bioreagents) buffer with 0.1% Tween 20. After blocking, the membrane was incubated with primary antibody in blocking buffer overnight at 4°C. Polyclonal anti-SLCO1B3 (HPA 004943, Sigma Prestige antibodies) produced in rabbit was used as a primary antibody in a ratio of 1/1000. After the overnight incubation with primary antibody, the membrane was washed five times with 1X TBS with 0.1% Tween 20. After washing, the membrane was incubated with secondary antibody for one hour at room temperature on a shaking platform. HRP-conjugated Goat Anti-Rabbit IgG (Sigma Aldrich) was used as secondary antibody in a ratio of 1/2000. After one hour of incubation, membrane was washed gently several times with adequate amount of TBS buffer containing 0.1% Tween 20. After this washing step, the membrane was imaged using a Li-COR ODYSSEY imaging system. The detectable signal on the membrane was developed by Cytiva Amersham ECL Prime western blotting Detection Reagents according to the manufacturer instructions.

For cell transplantation studies, cells were grown to 80% confluency and then labeled in cell culture media with 5.0 mM Gd-EOB-DTPA and 2 μg/ml indocyanine green (ICG) near infrared fluorescence dye for 1.5 hours, after which cells were washed. To validate labeling, an aliquot of cells was pelleted and T1 was measured at 7.0T (Bruker Biospec) by variable TR method with parameters: T1 RARE, TRs: 85, 395, 822, 1443, 2435, 7500 ms, TE 8 ms, RARE factor 2, resolution 200 × 200 × 500 μm. ICG cellular uptake was also validated using fluorescence microscopy (Biotek Cytation3). Non-transduced cells were also similarly incubated and imaged to serve as control cells. For transplantation, 5×10^6^ dual labeled (Gd-EOB-DTPA and ICG) OATP1B3-expressing cells were pelleted and resuspended in 50 μl PBS. Food-grade chicken hearts were used as a model. 1×10^6^ cells in 10 μl PBS was slowly injected free-hand into the left ventricular wall of chicken heart (n = 5) using a Hamilton syringe and 22 gauge needle. Injected hearts were immediately imaged at 7.0T by 3D T1-weighted gradient echo MRI with parameters: TR/TE: 30/2.5, 1 average, FA 60°, resolution 250 um, FOV 40×30×30 mm, 8 min acquisition. MR images were analyzed in PMOD. 3D volumes of interest (**VOIs**) were drawn in non-injected heart region, region outside the heart (noise) and the hyperintense area from the injected cells. Contrast-to-noise ratio (**CNR**) was calculated.

After MRI, hearts were processed for cryofluorescence tomography (**CFT**) (Xerra, EMIT) to validate the location of bright MRI signals. Hearts were frozen over dry ice then embedded vertically in OCT in a 7.5×9.5×5 cm mold. White light and fluorescence images (excitation 780 nm, emission 835 nm) were acquired with 30 μm in-plane resolution and 50 μm slice thickness. Images were combined into stacks using software from Emit and then visualized in PMOD to create maximum intensity projections.

## Results

**Figure 1:**
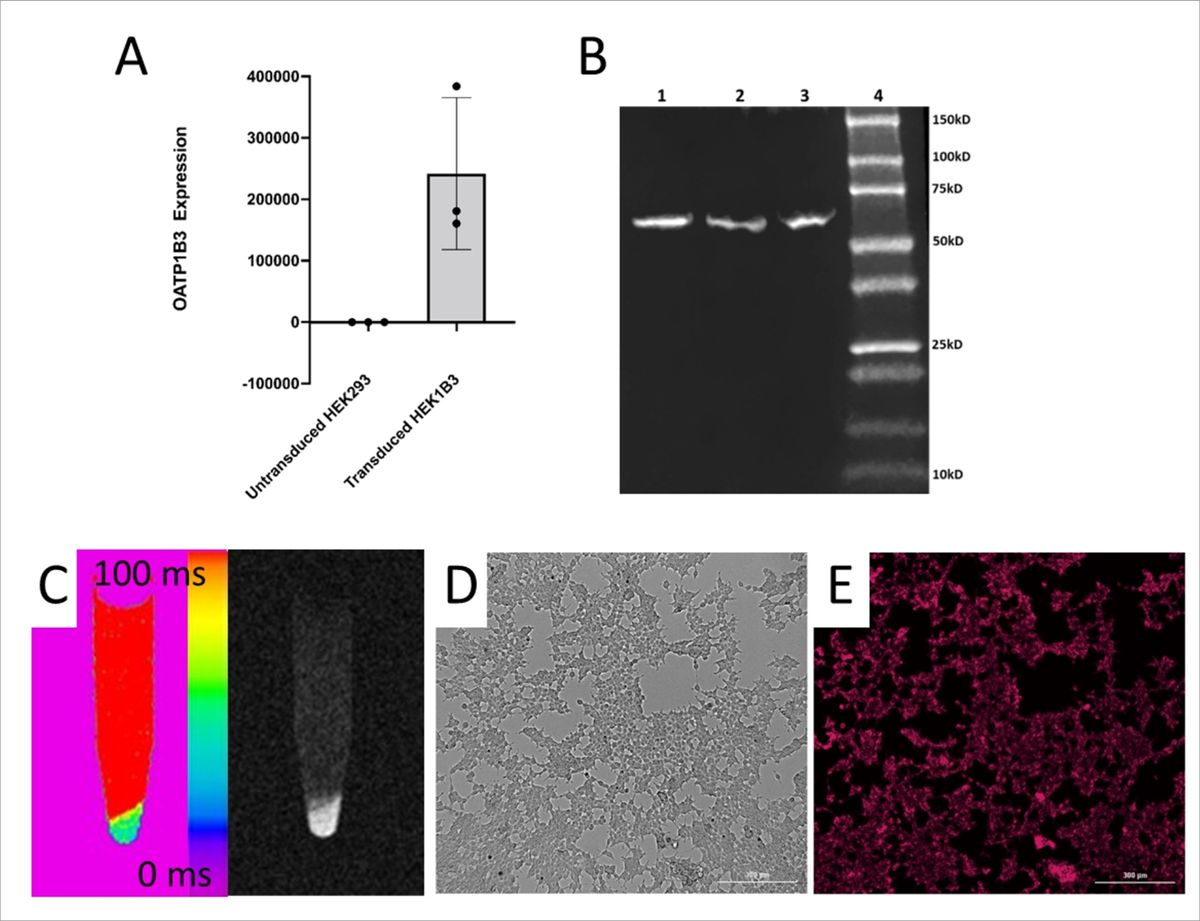
**A)** RT-qPCR of OATP1B3 gene expression in untransduced and transduced HEK293T cells. Y-axis is relative fold change in gene expression of 1B3; **B)** Western blot of OATP1B3 protein isolated from transduced HEK293T cells. Lanes 1–3 are separate cell preparations, Lane 4 is Precision Plus Protein Standard; **C)** T_1_ map and corresponding MRI of OATP1B3-expressing cell pellet and PBS supernatant in an Eppendorf tube following incubation in 5.0 mM Gd-EOB-DTPA and 2.0 μg/ml ICG for 1.5 hours. **D,E)** White light (D) and NIR fluorescent image (E) of OATP1B3-expressing cells harboring intracellular ICG following labeling. Scale bar is 300 μm.

Stable OATP1B3 expression in HEK293 cells was confirmed by qRT-PCR (Fig. 1A), Western blot to stain for OATP1B3 protein (Fig. 1B), and phenotypically by uptake of Gd-EOB-DTPA and ICG in transduced cells. T1 time for OATP1B3-expressing cells were 52 ms following incubation in Gd-EOB-DTPA (Fig. 1C), while the T1 time for non-expressing cells was ~ 1500 ms. ICG uptake into OATP1B3-expressing cells was verified microscopically (Fig. 1D–E), while control cells had no fluorescence.

**Figure 2:**
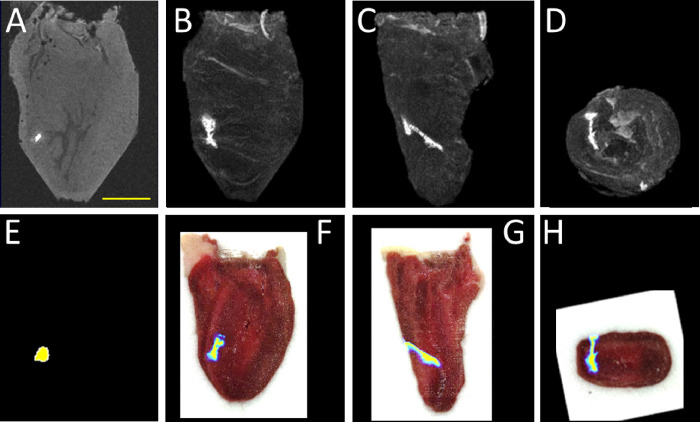
**A,E)** MRI of chicken heart with bright contrast spot from Gd-EOB-DTPA labeled OATP-expressing cells (A) with corresponding ICG NIR fluorescence from CFT image at same location (E). **B-D)** Maximum intensity projection orthogonal views from MRI of entire heart showing bright contrast from Gd-EOB-DTPA labeled OATP-expressing cells. **F-H)** ICG NIR fluorescence from CFT image (in rainbow color scheme) overlayed on white light image reconstructed to correspond to MRI views in (B-D). Some minor geometric distortion of CFT images relative to MRI is visible due to freezing and repositioning the heart for CFT. Scale bar = 1 cm.

**Figure 3:**
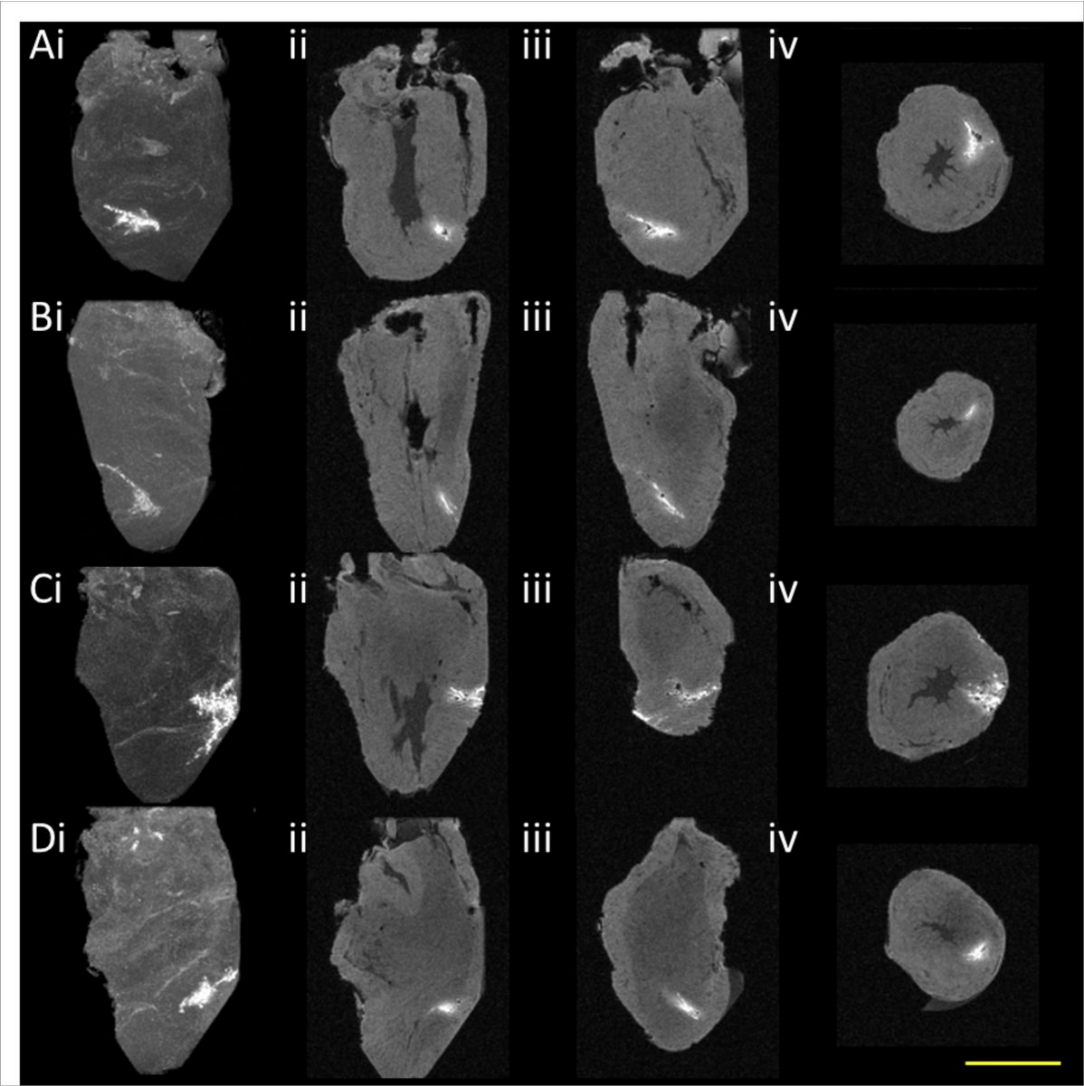
Cell transplant detection in 4 chicken hearts **(A-D).** Maximum intensity projections **(i)**and 3 plane views **(ii-iv)**. Scale bar = 1 cm.

Following injection of Gd-EOB-DTPA and ICG labeled cells into the hearts, these cells appeared as hyperintense signals on T1-weighted MRI (Fig. 2A). Maximum intensity projections show the extent of the cell transplant (Fig. 2B–D). The calculated volume of cells generated from MRI VOIs was 6.7 +/− 2.3 μl, close to the intended 10 μl injected volume, with CNR 38.4 +/− 15.4. CFT validated near identical overlap of injected cells both as single slices (Fig. 2E) and as projections (Fig. 2F–H), verifying that the bright MRI signal was generated from the ICG-labeled transplanted cells. Figure 3 shows 4 additional chicken hearts injected with Gd-EOB-DTPA labeled OATP1B3-expressing cells. Taken together, these multi-modal imaging data show that transplanted cells are not only deposited at the intended site of delivery, but also fill the injection path. These data further underscore the heterogeneity of cell transplantation, albeit these results are from a specific in vitro scenario.

## Discussion

Hepatic OATPs are poised to make a major impact for molecular imaging by MRI due to their ability to transport FDA-approved MRI contrast agents into cells that express the transporters. We add to this growing field by demonstrating that OATP1B3-expressing cells can be pre-labeled by incubation with Gd-EOB-DTPA prior to injection, and their location immediately post-transplantation can be readily detected as hyperintense signal by clinically familiar T1-weighted MRI. As non-hepatocytes do not normally express these hepatic transporters, one would need to either transiently transfect or stably transduce cells to express OATPs prior to use. These molecular biology methods are facile and standard, yet it does add an extra step in cell preparation.

No study more poignantly proved the necessity to image cell transplants immediately after delivery than de Vries, et al ^26^. Here, dendritic cells were (meant to be) injected into lymph nodes of patients as a potential cancer vaccine. Cells were labeled with iron oxide nanoparticles to enable their visualization via T2-weighted MRI, and MRI identified that ~ 50% of cell injections had been erroneously injected into fat, rather than lymph nodes. Iron oxide labeling yields very high sensitivity, with single cell detection limits under some circumstances ^2^, but the contrast obscures underlying anatomy ^27–28^ and in the case of the delivery of a clinically relevant dose of millions of cells, the contrast fully obscures the signal from the underlying and directly adjacent anatomy due to the well-known ‘blooming artifact’. Indeed, in the de Vries paper, it would have been challenging and potentially impossible to discriminate whether cells were injected successfully just inside the lymph node or unsuccessfully just outside the lymph node. As such, one potential advantage of using Gd-EOB-DTPA for cell labeling and MRI-based cell tracking is the use of T1-weighted MRI to generate hyperintense signals, rather than T2/T2*-weighted MRI to generate anatomy-obscuring dark contrast. The MRI in Figs. 2 and 3 show the full extent of cell transplants at high resolution, and without confounds from dark contrast artifacts.

Another potential advantage of this paradigm is the likelihood of contrast agent clearance from the transplantation area in the case of cell death. We hypothesize this to be the case as the small Gd-EOB-DTPA molecule would quickly diffuse and other cells would not accumulate it, even macrophages, due to the lack of OATP transporters. Indeed, the bystander effect from macrophage accumulation of iron oxide nanoparticles from dead cells is a major confounder for MRI-based cell tracking using iron oxide particles^29^. Lastly, this method is unlikely to impact the ability to later re-locate these transplanted cells by the established method of IV injection of Gd-EOB-DTPA or Gd-BOPTA as studies have shown that the agent clears from cells between 1 and 5 days^6–12^.

## Conclusion

MRI-based cell tracking of OATP-expressing cells immediately following transplantation is robust and feasible by pre-labeling cells with Gd-EOB-DTPA. Detection can be accomplished using T1-weighted MRI, creating bright contrast, as opposed to T2 and T2*-based dark contrast methods. The use of FDA-approved MRI contrast agent with clinically familiar imaging protocols may hasten the use of MRI-based cell tracking in research and development, and clinical monitoring, of cell therapy.
